# The quality of life in patients treated for rectal cancer

**DOI:** 10.1186/1471-2318-11-S1-A27

**Published:** 2011-08-24

**Authors:** M Mangiapane, EV Bonafede, G Di Carlo, C Lo Piccolo, M Vitrano, G Diana

**Affiliations:** 1 U.O.C. Chirurgia Generale e Geriatrica, AOU Policlinico di Palermo, Palermo, Italy

## Background

The aim of this study is to investigate the quality of life (QOL) in patients treated surgically for rectal cancer. We will evaluate different surgical treatments, complications, presence and absence of a protective or definitive stoma and how this can influence the patient’s quality of life.

## Materials and methods

We have evaluated 69 consecutive patients (39 male and 30 female) operated for rectal cancer in our ward. The preoperative investigation includes, according to guidelines for CRC treatment: pancolonscopy, chest radiography and a CT scan of the abdomen. The most appropriate surgical treatment was chosen depending on the results of the preoperative study (Table 1).

**Table 1:** 

	n	%
**Male**	39	55
**Female**	30	45
**Median age (years)**	68,6	45-92

A standard questionnaire investigating the quality of life was administered to all the patients in the preoperative time (t0), in the early postoperative time(t1) and 3 (t2), 6 (t3), 9 (t4) and 12 (t5) months after the operation. Our questionnaire, the same as EORTC QLQ-C30 [1], QLQ-C38 [2] and SF-36 [3], is composed of the items described in Table 2.

**Table 2:** 

Questionnaire items
**Age**
**Staging**
**Surgical treatment**
**Presence of stoma**
**Resume of non-working activity**
**Body functions**
**Emotional functions**
**Sexual functions**
**Social relations**
**Global QOL**

## Results

All the patients enrolled in the study answered our questionnaire. 31 of the patients underwent anterior resection of the rectum with total mesorectal excision(ARR), 24 underwent lower anterior resection (Low ARR), 9 underwent ultra-low anterior resection (Ultra-low ARR), 1 underwent Hartmann resection, 1 underwent abdominoperineal resection sec. Miles and 3 patients were treated by endoscopical resection (Table 3).

**Table 3:** 

Surgical Treatment	n	%
**ARR**	31	45
**Low ARR**	24	34.8
**Ultra-low ARR**	9	13
**Others**	5	7.2

A temporary stoma was made in 32 patients, and a definitive one in 2 patients. The stoma was made only in the patients with an elevated risk of anastomotic leakage. The overall complication rate was 20.2%, interesting 14 patients of the total as described in the table 4.

**Table 4:** 

	N° patients	%
**Anastomotic leakage**	10	14.4
**Fistula**	3	4.3
**Anastomotic stenosis**	1	1.4
**Total**	14/69	20.2

**Figure 1:**
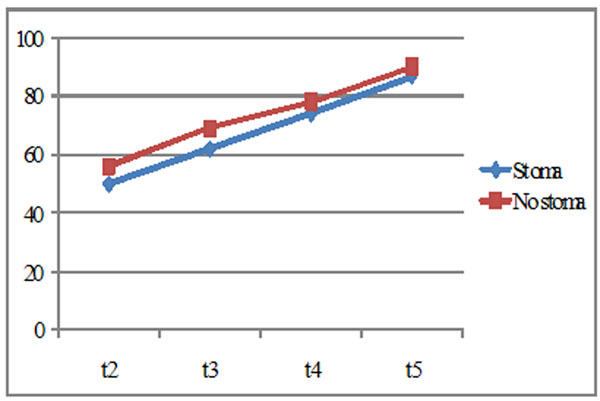


The patients, in particular those with stoma, have a decrease of the QOL global index in respect to self image and social life. In t2, t3, t4, t5 the patients have a gradual improvement of their QOL although the patients with stoma always present a lower score (Fig. 1).

## Conclusions

The perception of quality of life is a dynamic reality that changes according to the length of time we evaluate the patient. Our study, in agreement with scientific literature [4-6], confirms that quality of life increases with time and that although rectal cancer and its surgical treatment may produce functional and psychological deficit the QOL remains elevated.

## References

[B1] Aronson Nk Ahmedzai S The European Organization for Research and Treatment of Cancer QLQ-C30: a quality of life instrument for use in international clinical trials in oncology J Natl Cancer Inst 1993 85 365 376 10.1093/jnci/85.5.365 8433390

[B2] Sprangers Ma Te Velde A The construction and testing of the EORTC colorectal cancer specific quality of life questionnaire module (QLQ-CR38). European Organization and Treatment of Cancer Study Group on Quality of Life Eur J Cancer 1999 35 238 247 10.1016/S0959-8049(98)00357-8 10448266

[B3] Ware Je Sherbourne Cd The MOS 36-item short-form health survey (SF 36) 1: conceptual framework and item selection Med Care 1992 30 473 483 10.1097/00005650-199206000-00002 1593914

[B4] Ross L Abild-Nielsen Ag Quality of life of Danish colorectal cancer patients with and without a stoma Support Care Cancer 2007 15 505 513 10.1007/s00520-006-0177-8 17103196

[B5] Carlsson E Berndtsson I Hallen Am Lindholm E Persson E Concerns and Quality of Life Before Surgery and During the Recovery Period in Patients With Rectal Cancer and an Ostomy J Wound Ostomy Continence Nurs 2010 Nov 3. [Epub ahead of print] 10.1097/WON.0b013e3181f90f0c 21052026

[B6] Fucini C Gattai R Urena C Bandettini L Elbetti C Quality of life among five-year survivors after treatment for very low rectal cancer with or without a permanent abdominal stoma Ann Surg Oncol 2008 15 1099 1106 10.1245/s10434-007-9748-2 18181002

